# Single-Cell RNA Sequencing of the Adult Mammalian Heart—State-of-the-Art and Future Perspectives

**DOI:** 10.1007/s11897-021-00504-3

**Published:** 2021-02-25

**Authors:** Monika M. Gladka

**Affiliations:** 1 Hubrecht Institute, Royal Netherlands Academy of Arts and Sciences (KNAW) and University Medical Centre, PO Box 85164, 3508 AD Utrecht, The Netherlands; 2 Present Address: Department of Medical Biology, Amsterdam University Medical Center, Amsterdam, The Netherlands

**Keywords:** Single-cell RNA sequencing, Heart, Cardiomyocytes, Cardiac disease

## Abstract

**Purpose of the Review:**

Cardiovascular disease remains the leading cause of death worldwide, resulting in cardiac dysfunction and, subsequently, heart failure (HF). Single-cell RNA sequencing (scRNA-seq) is a rapidly developing tool for studying the transcriptional heterogeneity in both healthy and diseased hearts. Wide applications of techniques like scRNA-seq could significantly contribute to uncovering the molecular mechanisms involved in the onset and progression to HF and contribute to the development of new, improved therapies. This review discusses several studies that successfully applied scRNA-seq to the mouse and human heart using various methods of tissue processing and downstream analysis.

**Recent Findings:**

The application of scRNA-seq in the cardiovascular field is continuously expanding, providing new detailed insights into cardiac pathophysiology.

**Summary:**

Increased understanding of cardiac pathophysiology on the single-cell level will contribute to the development of novel, more effective therapeutic strategies. Here, we summarise the possible application of scRNA-seq to the adult mammalian heart.

## Introduction

In the past few years, single-cell sequencing has become a very powerful method to study gene expression changes in different organs [1, 2]. Even though the application of this technique to many tissues appeared to be fairly straightforward, the use of this method in the heart seemed to be quite challenging [3]. This is probably because of the unique nature of the heart tissue. On the one hand, cardiomyocytes are large and can be easily damaged by the enzymatic digestion and sorting, and on the other hand, nonmyocytes are smaller and more resistant and thus need more harsh treatment. Also, while studying a diseased heart, the pathological remodelling will have an impact on the procedure. For instance, during ischemic injury of the heart, there is formed a rigid fibrotic scar that is very difficult to dissociate into the single-cell suspension, often resulting in a poor RNA quality.

## Single-Cell RNA Sequencing, Why Do We Need It?

For decades, researchers all over the world have used traditional bulk sequencing methods to study gene expression changes in different tissues [4]. The obtained information was sufficient to study the total content of RNAs, including mRNA, rRNA, t-RNA, and noncoding RNA [5]. Undoubtfully, bulk RNA sequencing has greatly advanced our understanding of the molecular signalling involved in cardiac homeostasis and disease, but it has some limitations. The most significant advantage of single-cell sequencing over bulk sequencing is the ability to obtain the gene expression profile of individual cells. It can reveal complex and rare cell populations, uncover cell type–specific differences in gene expression, study heterogeneity within one cell type, and uncover new and potentially unexpected biological processes that could be overlooked with the traditional bulk sequencing [2].

**Fig. 1:**
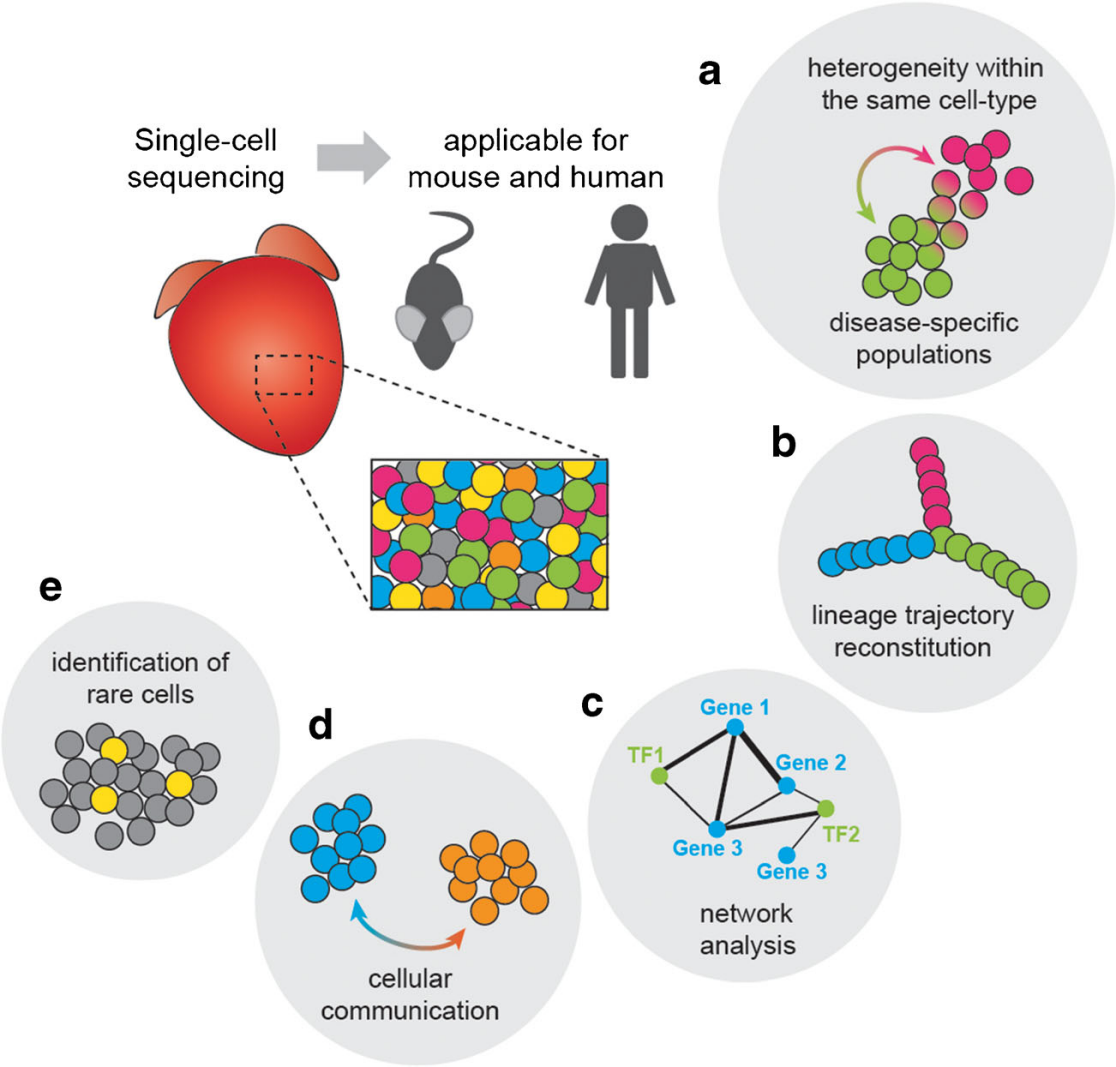
Schematic representation of a commonly used application of single-cell sequencing in the adult mammalian heart. **a** Heterogeneity within the same cell type, including disease-specific populations. **b** Lineage trajectory reconstitution. **c** Network analysis. **d** Cellular communication. **e** Identification of rare cells

The very first study using scRNA-seq was described in 2009 in Nature Methods by Tang and colleagues [6]. They used a labour-intense and time-consuming protocol to manually pick a single oocyte or blastomere under the microscope, followed by individual processing. Luckily in the following years, the scRNA-seq methods were much improved, increasing the stability and reproducibility and, at the same time, reducing costs and workload [1]. Microfluidics and robotics-based approached supported simultaneous isolation of thousands of cells, followed by rapid RNA extraction and downstream processing for sequencing [7–9]. Also, developments of more elaborate analysis methods led to a significant increase in studies using scRNA-seq. The first reports of using scRNA-seq in the heart came in 2016 from Sean Wu and Christine Seidman’s labs [10, 11]. They used scRNA-seq to profile the transcriptome of the mouse embryonic heart and identify lineage-specific gene programs that underlie early cardiac development. After that, in 2018, the first studies were published using scRNA-seq on adult healthy and diseased hearts [12–14]. Nowadays, multiple researchers use this technique in various cardiac disease models and even applied it to human tissue. The use of scRNA-seq in the cardiovascular field is continuously expanding, providing new detailed insights into cardiac pathophysiology. Here, we discuss the most commonly used application of scRNA-seq to the adult mammalian heart (Fig. 1).

## Studying the Heterogeneity Within the Same Cell Type, Including Disease-Specific Populations

One of the first studies that applied scRNA-seq to healthy and diseased hearts observed heterogeneity within the same cardiac cell type [14]. The authors found that there is heterogeneity in the cardiomyocyte population and that only a subset of them expresses myozenin 2 (*Myoz2*). MYOZ2 is a sarcomeric protein that tether a-actinin to calcineurin, a well-known driver of cardiac hypertrophy, therefore preventing the pathological hypertrophic response of cardiomyocytes [15]. MYOZ2 is expressed in the subset of cardiomyocytes close to the epicardial region of the heart, suggesting region- and function-dependent differences in the expression already in the healthy tissue. Another report using scRNA-seq aimed to investigate the heterogeneity among adult cardiomyocytes under baseline and pressure overload-induced cardiac hypertrophy [16]. They specifically focused on determining the transcriptional profile of mono- and multi-nucleated cardiomyocytes. In this study, Yekelchyk and colleagues reported that adult rod-shaped cardiomyocytes are homogenous and that mono-, bi-, and multi-nucleated cells express a nearly identical set of genes, which was an unexpected observation. In contrast, induction of hypertrophy induced significant transcriptional changes in cardiomyocytes and generated substantial heterogeneity [16]. Cardiac fibroblasts are another population that displays extensive transcriptional heterogeneity in the heart. In the manuscript by Vidal et al., they used snRNA-seq to investigate the impact of ageing on cardiac cells [17]. They discovered that aged fibroblasts displayed the most pronounced differential gene expression pattern among all cell types, including dysregulation of inflammatory, extracellular matrix, angiogenesis, and osteogenic-related genes [17]. The study by Gladka et al. reported a disease-specific subpopulation of fibroblasts, and cytoskeleton-associated protein 4 (*Ckap4*) was identified as a novel marker specifically expressed in activated fibroblasts [14]. Single-cell transcriptome analysis was also used to generate the expression atlas of resident cardiac endothelial cells that promote neovascularization following ischemic injury [18]. Li et al. demonstrated that the structural integrity of adult cardiac endothelium post-myocardial infarction is mainly sustained through a clonal proliferation of resident endothelial cells [18]. By defining region-, age-, or disease-specific in different cell populations and corresponding gene expression signature, we might be able to identify novel molecular mechanisms relevant for cardiac disease and new therapies.

## Studying Cellular Communication Between Cardiac Cell Types

Characterisation of the cardiac interaction network of cells form the heart is necessary for understanding cardiac homeostasis and disease. In 2018, Skelly and colleges reported that scRNA-seq is a great tool to study cellular communication in the heart. They performed scRNA-seq on a noncardiomyocyte fraction from a mouse heart, and based on a previously published dataset from humans [19], they generated a list of potential ligand-receptor pairs and applied it to their mouse scRNA-seq data. The extensive analysis revealed a dense intercellular communication network between all cardiac cell types, with fibroblasts being the most communicative cells [13]. Farbehi et al. also constructed a cell-to-cell communication network of ligand and receptor interactions based on the previously mentioned human ligand-receptor pair dataset [19]. They performed scRNA-seq on the noncardiomyocyte fraction of the healthy and injured heart. The obtained data showed dynamics in myeloid and fibroblast lineage post-myocardial infarction and provide insights for the analysis of cardiac homeostasis, inflammation, fibrosis, and regeneration. They also investigated a previously undescribed fibroblast lineage trajectory present in both sham and ischemic hearts [20]. Most cells express tens to hundreds of ligands and receptors to create a highly connected signalling network by multiple ligand-receptor pairs, yet the functional readout of this interaction on target gene expression in the recipient cells is not fully understood. Browaeys et al. [21] recently developed a tool called NicheNet for modelling intercellular communication on the single-cell level by linking ligands to target genes. By doing so, it is not only possible to model how gene expression of one cell is influenced by interaction with another cell, but also which signalling mediator may be involved [22].

## Network and Trajectory Analysis

Analysis of network and trajectory is tightly connected to cell-to-cell communication, but it requires more extensive data analysis. Researchers use these analyses to define the pattern of a dynamic process encountered by single cells, based on their gene expression, and then organise cells depending on their progression through that process [23]. Weighted gene co-expression network analysis (WGCNA) is a commonly used data mining method for studying biological networks based on pairwise correlations between variables [24]. In the study of Nomura et al., they applied WGCNA to their single-cardiomyocyte transcriptomes and identified several co-expression gene modules [12]. This was followed by Monocle analysis which allows studying how cells choose between one of several possible end states [25, 26]. They found that after the reconstitution, the branched trajectory of cardiomyocytes from pressure overload mouse model was able to distinguish gene modules for cardiomyocyte hypertrophy and failure. The extensive analysis showed that during the early hypertrophic response in cardiomyocytes, there is an activation of mitochondrial genes closely correlated with ERK1/2 and NRF1/2. On the other hand, persistent overload leads to bifurcation into adaptive and failing stage via p57 signalling [12]. Another study used a trajectory analysis to investigate the molecular signature at different stages during the progression of pressure overload-induced cardiac hypertrophy. In the manuscript by Ren et al., it was reported that macrophage activation is a crucial factor for the development of cardiac hypertrophy, indicating cellular cross-talk between different cardiac cell types. The authors also showed that macrophage activation and subtype switching was successfully targeted by therapeutic intervention [27]. Wang et al. were able to construct cellular interactomes and regulatory signalling networks at multiple postnatal stages in the mouse heart [28]. In-depth analysis of the cellular network identified fibroblasts as a critical constituent in the microenvironment, promoting cardiomyocyte maturity. They also provide insights on how the manipulation of cardiomyocyte maturity impacts disease development and cardiac regeneration [28]. Another study by See et al. employed microfluidic flatform for single nuclear RNA-seq (snRNA-seq) to the frozen mouse and human failing and nonfailing adult hearts and revealed significant cellular heterogeneity [29]. They characterised these subgroups by WGCNA and discovered long intergenic noncoding RNAs (lincRNAs) as key nodal regulators. Knockdown of nodal lincRNAs affected the expression levels of genes related to dedifferentiation and proliferation within the same gene regulatory network. Additionally, they found that subpopulations of cardiomyocytes upregulate cell cycle–related genes, suggesting a unique endogenous regenerative potential [29]. There is no doubt that understanding how gene expression programs are controlled involves identifying regulatory interactions between transcription factors and target genes [30]. Numerous trajectory reconstitution algorithms are used to study the gradual transcriptomic response of cells to disease over time [12, 31]. Moreover, the application of WGCNA can characterise a network of co-expressed genes and together drive biological or pathological processes. Applying these analyses to the single-cell dataset not only can significantly advance the understanding of the processes driving cardiac pathologies but also shed light on new strategies for cell type- and stage-specific therapeutic interventions.

## Identification of Rare Cells in the Heart

scRNA-seq allows us to characterise rare cell populations that are often overlooked in bulk sequencing approaches. Identifying rare cells is crucial to acquire a better understanding of normal or diseased tissue biology. To address this challenge, Grün et al. developed a RaceID algorithm suitable for rare cell type identification in a complex population of cells. They applied it for the first time to the intestine and identified regenerating islet-derived protein 4 (*Reg4*) as a novel marker for enteroendocrine cells, a rare population of hormone-producing intestinal cells [32].

Regenerative properties of the adult mammalian heart are insufficient to repair the heart post-ischemic injury. Many researchers seek to find evidence of cardiomyocyte renewal in the adult heart. Kretzschmar et al. attempted to look for resident cardiac stem cells (CSCs) that could contribute to cardiac regeneration [33]. Since the existence and significance of such cells in the heart are highly debated, they used scRNA-seq followed by the RaceID algorithm analysis and genetic lineage tracing to search for these rare cells. Unfortunately, they found no evidence for the existence of a quiescent CSC population or de novo generated cardiomyocytes that could contribute to cardiac repair [33]. Another study by Zhang and colleagues used multi-reporter mouse models and snRNA-seq to study cardiomyocyte dedifferentiation and proliferation. The obtained data suggest that increased endogenous cardiomyocyte renewal in post-ischemic hearts arises from the dedifferentiation and proliferation of pre-existing cardiomyocytes and not by cardiac differentiation of putative adult CPCs [34]. They pointed out that even prolonged cardiomyocyte dedifferentiation and subsequent proliferation can adversely affect cardiac function [35]. Thus, additional knowledge of specific regulators of both dedifferentiation and cell cycle reactivation will be required to be able to promote endogenous cardiomyocyte proliferation without diminishing heart function. More ongoing studies aim to find and characterise the rare population of proliferative cardiomyocytes that may explain why this process is so inefficient in the adult heart and perhaps how it can be manipulated for therapeutic benefit.

## Application for Human Cardiac Tissue

Naturally, to confirm the relevance of all discoveries related to cardiac diseases performed in mouse models, they must be validated in human samples. Unfortunately, accessibility to fresh human cardiac tissue is not always possible. Biopsies from patients suffering from cardiac diseases could be a valuable option but to obtain cardiac tissue from healthy individuals is nearly impossible. Despite these challenges, several studies managed to apply scRNA-seq to the adult healthy and diseased human heart. In the manuscript of Nomura et al., they performed sequencing on intact cardiomyocytes manually isolated from healthy individuals and patients with dilated cardiomyopathy. They were able to validate the conservation of pathological transcriptional signatures that they previously identified in mouse [12]. In the study by Wang et al., the authors performed a comparative analysis of atrial and ventricular cells from healthy, failing, and partially recovered adult human hearts. They revealed the presence of cellular heterogeneity in cardiomyocytes and compartment-specific properties of the noncardiomyocyte fraction. Additionally, the authors showed that based on the cellular interaction network, cardiomyocytes’ contractility and metabolism are the essential features affected by cardiac function deterioration [36]. Commonly used droplet-based scRNA-seq systems (e.g., Drop-seq) are not applicable for intact adult human cardiomyocytes because of their large size. Therefore, many studies utilise frozen nuclei instead of intact cells for droplet-based systems. These approaches have high throughput and are highly suitable for frozen cardiac tissues stored in biobanks, which are much more accessible than fresh tissue. Selewa et al. applied Drop-seq technology to frozen nuclei from human heart tissue (DropNuc-seq) and demonstrated that this method could be successfully applied to identify different cardiac cells from frozen tissue [37]. The recent study of Litvinukova et al. used scRNA-seq and snRNA-seq to provide comprehensive transcriptomic data on six distinct cardiac regions of the adult human heart. Similar to other reports, they observed the cellular heterogeneity of cardiomyocytes, pericytes, and fibroblasts, and additionally, they analysed cell-to-cell interactions [38]. Microfluidic was also used by Tucker et al. to perform snRNA-seq of healthy human donors to identify and study the cellular and transcriptional diversity of the nonfailing human heart [39]. Hence, extensive analysis of the sequencing data from healthy adult human hearts on the single-cell level provides a comprehensive dataset of human cardiac cells and increases our understanding of cardiac biology.

## Proper Platform Selection for the Best Results

Dispersing and sorting cardiac tissue into single cell are crucial steps for successful scRNA-seq. Unfortunately, there are considerable limitations for all available methods to dissociate and sort cells, especially important when selecting large adult mouse or human cardiomyocytes. Manual cell picking was employed in some studies [12], but this method is labour-intense and might be biased toward cells that are picked. Plate-based platforms like flow cytometry [13, 14, 33, 38] or integrated fluidic circuit [29] are commonly used techniques for large and fragile cells like cardiomyocytes, yet the number of cells that can be processed at once is limited. On the other hand, microfluidic platforms like 10x Genomics [17, 18, 20, 34, 38] or Drop-seq [37] are commercially available droplet-based platforms that allow for rapid profiling of thousands of individual cells. These microfluidic platforms are not suitable for cells bigger than 30 μm in diameter. Therefore, they are commonly used for smaller noncardiomyocytes or nuclei isolated from cardiomyocytes rather than intact cardiomyocytes. ICELL8 [16, 27, 28, 36] systems also enable high-throughput processing of hundreds of single cells without the typical microfluidic cell size restrains or the imaging limitations of droplet-based systems. Thereby, a careful selection of the most suitable platform that fits our needs is a crucial step for successful single-cell transcriptomic analysis.

## Conclusions

**Fig. 2:**
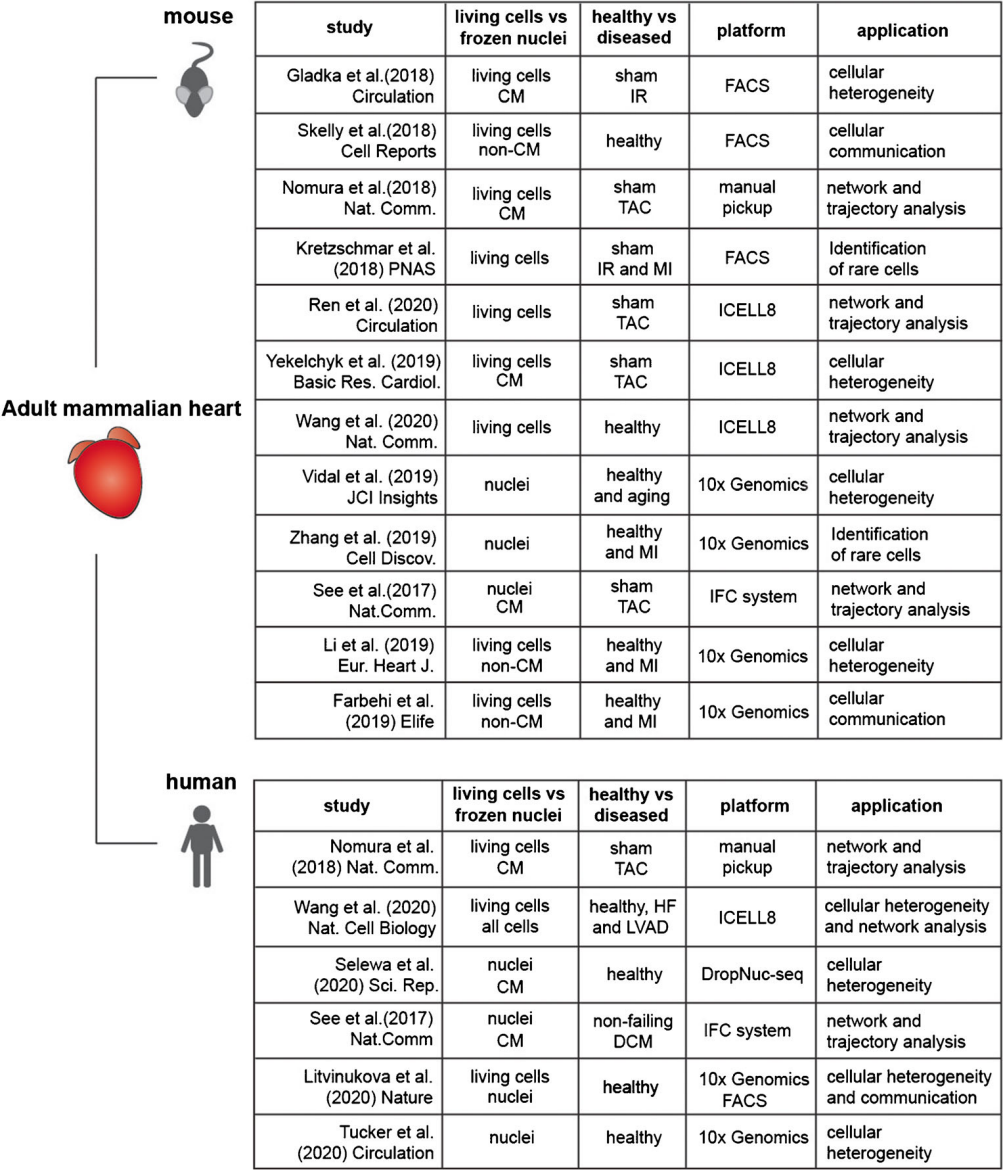
Overview of single-cell sequencing studies applied to adult healthy and diseased mouse or human heart. CM cardiomyocytes, IR ischemia/reperfusion, MI myocardial infarction, TAC transaortic constriction, HF heart failure, LVAD left ventricular assist device, DCM dilated cardiomyopathy

The exceptional resolution and valuable data obtained by scRNA-seq studies summarised here and by others (Fig. 2) continue to provide a new perspective into cardiac biology of healthy and diseased mammalian hearts. Increased understanding of molecular signalling specific for cardiac repair and regeneration [29, 33, 34], intercellular communication networks [12, 13, 20, 27–29], rare cell populations [33, 34], and new endogenous markers and mechanistic regulators of disease-associated processes [12, 14] will contribute to the development of novel, more effective therapeutic strategies. Perhaps in the future, combining scRNA-seq with spatial transcriptomics will further reveal spatial gene expression patterns in the single-cell resolution.
